# Investigation of Localized States in GaAsSb Epilayers Grown by Molecular Beam Epitaxy

**DOI:** 10.1038/srep29112

**Published:** 2016-07-06

**Authors:** Xian Gao, Zhipeng Wei, Fenghuan Zhao, Yahui Yang, Rui Chen, Xuan Fang, Jilong Tang, Dan Fang, Dengkui Wang, Ruixue Li, Xiaotian Ge, Xiaohui Ma, Xiaohua Wang

**Affiliations:** 1 State Key Laboratory of High Power Semiconductor Laser, School of Science, Changchun University of Science and Technology, 7089 Wei-Xing Road, Changchun 130022, China; 2 Department of Electrical and Electronic Engineering, Southern University of Science and Technology of China, Shenzhen, Guangdong 518055, China

## Abstract

We report the carrier dynamics in GaAsSb ternary alloy grown by molecular beam epitaxy through comprehensive spectroscopic characterization over a wide temperature range. A detailed analysis of the experimental data reveals a complex carrier relaxation process involving both localized and delocalized states. At low temperature, the localized degree shows linear relationship with the increase of Sb component. The existence of localized states is also confirmed by the temperature dependence of peak position and band width of the emission. At temperature higher than 60 K, emissions related to localized states are quenched while the band to band transition dominates the whole spectrum. This study indicates that the localized states are related to the Sb component in the GaAsSb alloy, while it leads to the poor crystal quality of the material, and the application of GaAsSb alloy would be limited by this deterioration.

Gallium Arsenide (GaAs)-based III-V group material systems have been extensively investigated over several decades for optoelectronic applications^1^,^2^,^3^,^4^,^5^. Band gap tailoring of GaAs-based materials is an important task for hetero-epitaxial devices. Specifically, the incorporation of small fractions of antimony (Sb) into GaAs-based materials results in a significant reduction of the band gap^6^,^7^,^8^,^9^, which demonstrates the potential for mid-infrared range of electronic and optoelectronic applications, particularly for edge-emitting lasers^10^ and vertical cavity surface emitting lasers (VCSELs)^11^. To be specific, GaAsSb alloy can be applied in data-communication lasers in the range of 1.3–1.5 μm^12^,^13^,^14^,^15^,^16^,^17^ and GaInAs/GaAsSb multi-quantum well (MQWs) have been used as the gain medium for 2–3 μm type-ІІ MQWs laser^18^. On the other hand, GaAsSb materials can be used for solar cell because their wide light absorption across the wavelength of solar radiation^19^,^20^,^21^, and infrared photodetectors applications^22^,^23^,^24^. Furthermore, the bandgaps can be achieved by varying Sb alloy composition in GaAsSb, which is internally lattice matched with InP-based devices^25^.

However, in spite of the GaAsSb alloy’s potential applications, very little work has been done on the optical properties related to bulk GaAsSb materials^6^,^7^,^26^,^27^, namely, comprehensive spectroscopic characterization at low temperature range. The information about carrier dynamics, optical transition and their temperature dependent near band edge transitions properties are also scarce. In this work, we have grown composition dependent GaAsSb epilayers on GaAs substrate by molecular beam epitaxy (MBE) and investigated their carrier dynamics and optical properties. The Sb component dependent alloy and their localized degrees are discussed. Our results show that both localized and delocalized states can be found in all the materials, while the degree of localized states are related to the Sb mole fractions. We also note the degeneration of GaAsSb alloy quality under higher Sb incorporation. Such information is important for their further applications.

## Results and Discussions

**Figure 1:**
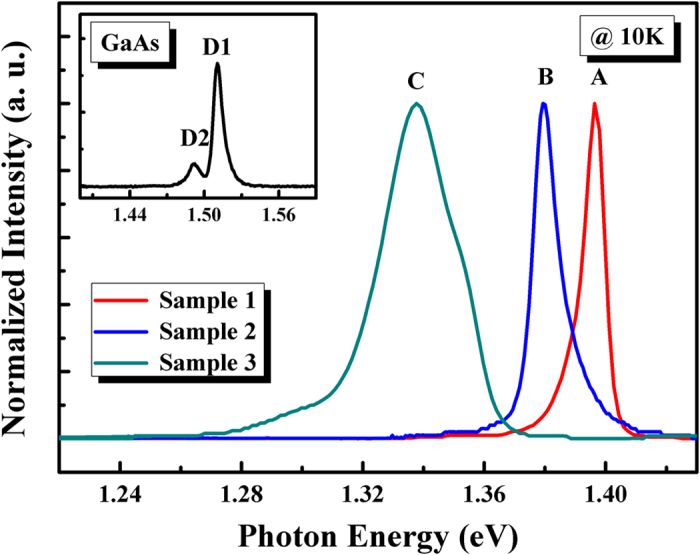
PL spectra of GaAsSb alloy samples measured at 10 K; The inset shows the PL spectrum of GaAs substrate at 10 K.

Figure 1 displays the low temperature (10 K) photoluminescence (PL) spectra of sample 1, 2, 3 and GaAs substrate (the inset of Fig. 1). The main peak positions of the GaAsSb samples and GaAs substrate are marked as A, B, C and D1, which were located at 1.396, 1.379, 1.338 and 1.510 eV, respectively. D2 (1.494 eV) has been attributed to band to acceptor (B-A) transition^28^. It can be clearly seen that the peak energy shows a red shift with the increase of Sb component. This phenomenon confirmed the existence of bandgap tailoring effect after the incorporation of Sb element inside the GaAsSb alloy. The full width of half maximum (FWHM) of the GaAsSb samples and GaAs substrate were 7.78, 9.99, 28.9 and 8.24 meV, respectively. Furthermore, the asymmetrical PL line shape suggests that the emission should have different radiative recombination mechanisms. It is interesting to note that the line shape of peak A (sample 1) showed a sharp high-energy cut-off and a low-energy tail. In contrast, peak B (sample 2) shows an opposite shape with a sharp low-energy cut-off and a high-energy tail. The peak C (sample 3) could be deconvoluted into 3 emission peaks (using Gaussian function, as shown in Supplementary Figure S1 and Table S1) because the peak shoulder exist on both side of the main peak. The samples show different emission characteristics, while it is difficult to reveal the hided mechanism only based on the emission at 10 K.

**Figure 2:**
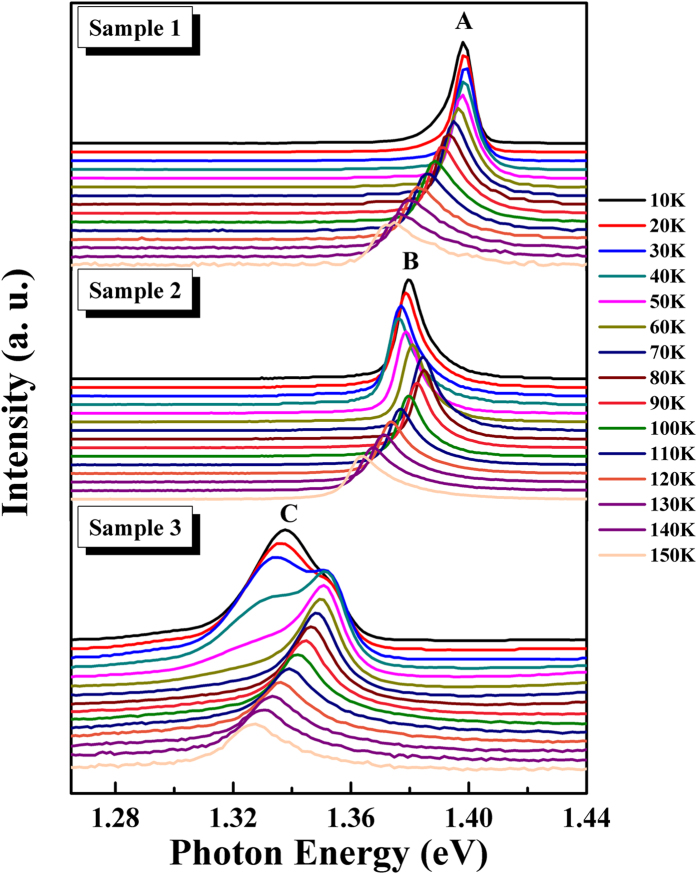
Temperature dependent PL spectra of sample 1–3 during the temperature range from 10 to 150 K.

In order to further investigate the effect of Sb on the optical properties of GaAsSb epilayer, temperature dependent PL measurement was carried out under a laser excitation around 80 mW^29^. The normalized PL spectra of the samples which were measured in the temperature range between 10 and 150 K are presented in Fig. 2. The curves of the temperature dependent spectra were all intentionally offset along the y-axis for better clarity. The peaks of the GaAsSb samples exhibit a redshift when Sb component increased, and the evolution of the emission peaks show significant different behavior. It is noted that the PL spectra exhibit a pronounced broadening at higher Sb component, which can be ascribed to the inhomogenous due to the Sb incorporation. In sample 1, the emission peaks (peak A) exhibit redshift with the increase of temperature. Peak A displays a low energy shoulder at 10 K, which was due to the distribution of carriers at different emission states. It is interesting that the PL spectra of sample 2 (peak B) exhibit a typical ‘S’ shape behavior^30^,^31^,^32^. The peak B had a trend of redshift first, and then blueshift, finally redshift again (at around 80 K) as the temperature continuously increases. This unusual temperature dependence of the PL peak significantly deviated from that predicted by Varshni or Bose-Einstein formula^33^, revealing the existence of localized states in the GaAsSb epilayers. The PL spectra of sample 3 exhibit a broadening peak, and two emission peak exist at 40 K. Based on the previous reported results^32^, the high energy peak is related to the band to band emission, and the low energy peak is related to localized state because it is far away from the conduction band edge.

To understand the physical mechanism behind the temperature dependent measurement, the detailed PL characteristics including emission peak energy and the FWHM of the GaAs substrate and GaAsSb epilayer samples were exampled.

**Figure 3:**
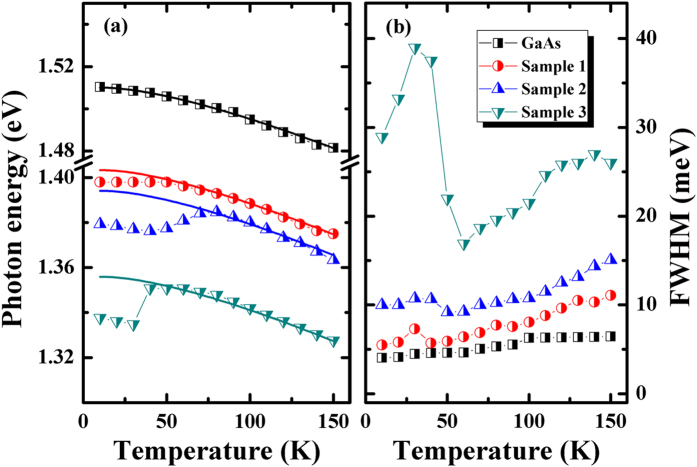
(**a**) Temperature dependent peak position of GaAs substrate and sample 1–3, where the colored solid lines are theoretical fittings; (**b**) temperature dependent FWHM of the GaAs sample 1–3.

In order to analyze the emission evolvement, the peak emission energies of GaAs substrate and sample 1–3 as a function of temperature are plotted in Fig. 3(a). The temperature dependent PL emission of GaAs substrate is shown in Supplementary Figure S2. The symbols in the figure are experimental data points while the solid lines represent the theoretical curves according to the Manoogian and Leclerc model^33^. The theoretical model is described as follow.

The temperature dependence of semiconductors band gap shrinkage can be well described by the following model shown as equation (1)^34^,^35^,^36^.


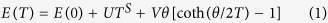


where *E*(0) is the band gap at 0 K, *U* is the lattice dilation coefficient, *S* describes the average exciton-phonon coupling strength, *V* is the temperature-dependent shift in the band gap, and *θ* is the temperature-related parameter. The temperature dependence of *E*(*T*) is due to both thermal expansion of the lattice and renormalization of the band energies by electron-phonon interaction.

Figure 3(a) shows the difference between experimental data and theoretical fitting in low temperature range (10–150 K), the energy deviation of the sample 1, 2 and 3 at 10 K were 5.3, 14.8 and 17.3 meV, respectively. The energy deviation could be ascribed to the localization energy of carriers at low temperatures. In our case, the degree of localization energy shows the linear relationship with the Sb component increased. It is indicated that the localized state originated from the Sb doped. Above 100 K, the plots were in coincidence with the theoretical curve when the temperature increases, carriers with sufficient thermal energy can readily be detrapped from the localized states and reveal more characteristics of band to band recombination.

Figure 3(b) presents the temperature dependent of FWHM parameters in GaAs substrate and sample 1–3. The FWHM of GaAs substrate shows the monotonic increasement with the temperature raises. However, we could observe the nonmonotonic behavior in GaAsSb alloy samples. The FWHM increases firstly, then decreased, and finally increase again. This behavior also confirmed the existence of the localized state in GaAsSb alloy samples.

**Figure 4:**
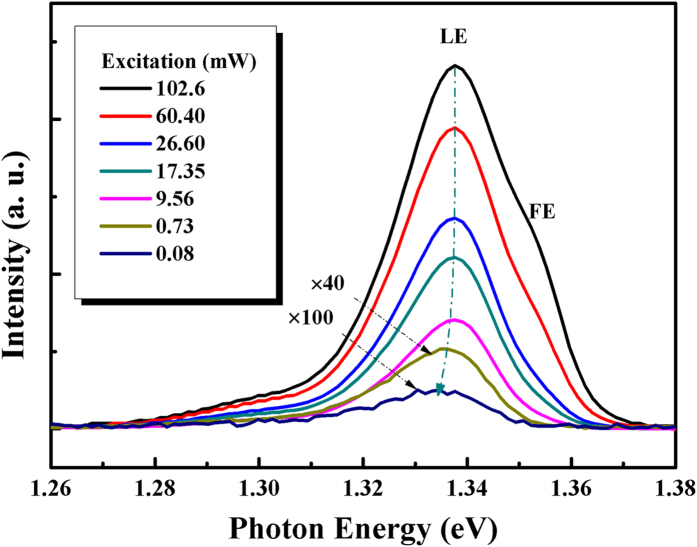
PL spectra of GaAsSb alloy sample 3 under different excitation intensities at 10 K (the selected excitation powers for comparison).

**Figure 5:**
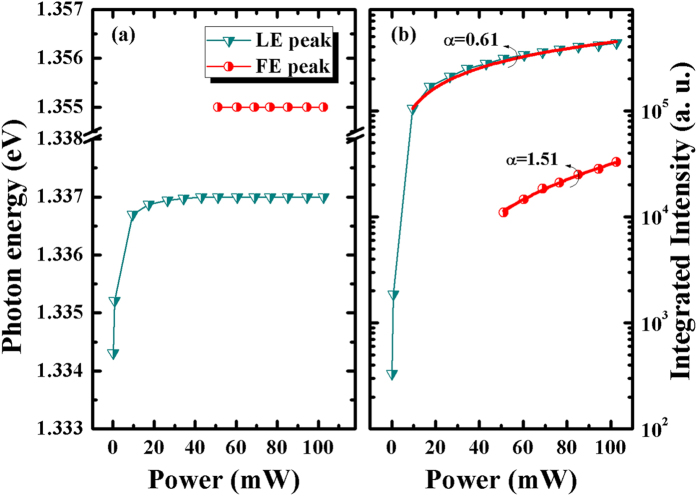
(**a**) The plots of sample 3 FE and LE peak positions under various laser excitation power, (**b**) the integrated FE and LE PL intensity under various laser excitation power (the solid lines represent the fitting curves of the Eq. (2)).

The PL peak position of GaAsSb alloy sample 3 exhibited a giant blueshift with the increase of laser excitation, as shown in Fig. 4. This blue shift was caused by the state filling of the localized states due to alloy potential fluctuations. It is known that in ternary alloy, the band edge emission always presents an exponential tail in the joint density of states (DOS) due to the random potential fluctuations caused by Sb incorporation. With the increase of excitation, localized states will be eventually saturated, and the band to band emission peak showed as a shoulder at the high energy side. It is deduced that the high energy shoulder is located at 1.355 eV, which is similar with the one in Fig. 3(a), therefore, it can be ascribed to free exciton (FE). The FE peak enhanced quickly with the increase of laser excitation. The peak located at 1.3376 eV could be ascribed to localized exciton (LE). To investigate the detail of the FE and LE emission evolution, we analyzed the peak positions and integrated PL intensity of FE and LE under various excitation powers, as shown in Fig. 5.

From Fig. 5(a), we can observe that the FE peak showed a slightly red shift with increasing the excitation energy, coincident with the thermal effect in semiconductors. The LE peak first showed a giant blue shift, this phenomenon was consistent with the saturate effect in Fig. 4.

Considering the integrated intensity of emission *I* can be expressed as equation (2):


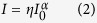


where *I*_*0*_ is the power of the excitation laser radiation, *η* is the emission efficiency and the exponent *α* represents the radiative recombination mechanism^37^,^38^. This expression is applied to the low excitation range^39^. In the Fig. 5(b), the symbols display the experimental data of the FE and LE, the red lines exhibit the fitting curves according to equation (2). With the increase of excitation power from 0.08 to 102.6 mW, the PL integrated intensity increase linearly, the parameter *α* of LE peak was 0.61(α < 1), which meant this emission is different from the band gap emission, and can be related to the impurity. The evolution of LE peak intensities exhibit exponential trend and saturated effect, and once again confirm that this peak comes from localized exciton emission. For FE peak, the value of α = 1.51 (1 < α < 2) can be obtained, which supports the excitonic recombination^37^,^38^,^39^,^40^.

## Conclusion

In conclusion, we have investigated the optical properties of the GaAsSb alloys by temperature and excitation intensity dependent measurements. The GaAsSb alloy showed the unique emission evolution at the low temperature range. The deviation of the emission energy in low temperature PL spectra was attributed to localized state in GaAsSb alloy. In the case of GaAsSb alloy sample 2, the “S” shape temperature dependent PL emission confirmed the existence of localized states the alloy. The plots of temperature dependent peak position confirmed that the degree of exciton localization raise with increasing of the Sb component. From the results, it is noted that the localized state become deeper with the Sb component increase, and this phenomenon results from the fluctuation of the Sb in the alloy. It is suggest that the crystal quality of GaAsSb alloy degeneration with the increasing of Sb component. Our results can be very meaningful in fully understanding of the mechanism of radiative emission in GaAsSb materials. Further, we consider the degree of localization can be a standard to evaluate the degeneration of alloy quality and the performance of the devices. Meanwhile, these results will be helpful to utilize GaAsSb materials and solve the limitation of GaAsSb devices.

## Experimental Methods

### Materials Epitaxy Growth

In this work, MBE was used to prepare the high-quality GaAsSb epilayers on semi-insulating GaAs substrates. The grown parameters are described as follows. The substrates were transferred into the grown chamber and first removed the oxides layer at 560 °C, then grown 100 nm GaAs buffer layer at 620 °C, finally grown 350 nm GaAsSb layer at 600 °C. The composition of antimony in the GaAsSb epilayer was controlled by antimony needle of antimony source. Table 1 shows the growth condition and Sb fraction of the samples. The AFM images were illustrated in Supplementary Figure S3. The Sb fraction was calculated by the PL spectra of the samples at 300 K (as shown in Supplementary Figure S4).

### Optical Measurements

For the PL measurement, the spectra were dispersed by HORIBA iHR550, and PL spectra were detected by an InGaAs detector. A 655 nm semiconductor diode laser was used as the excitation source. A standard phase lock-in amplifier technique was employed to enhance the signal-to-noise ratio. The excitation power of 80 mW and the spot area of the laser of about 0.4 cm^2^ were used during temperature dependent PL spectra measurement, and the measurement temperature in excitation dependent PL spectra is fixed at 10 K.

## Additional Information

**How to cite this article**: Gao, X. *et al*. Investigation of Localized States in GaAsSb Epilayers Grown by Molecular Beam Epitaxy. *Sci. Rep.* **6**, 29112; doi: 10.1038/srep29112 (2016).

## Supplementary Material

Supplementary Information

## Figures and Tables

**Table 1:** The growth condition of the GaAsSb alloy samples.

Sample	Growth Temperature	As/Sb Beam Ratio	Sb Component
1	600 °C	28:1	6%
2		16:1	8%
3		7:1	9%
