# Supplementary material for: Investigation of Localized States in GaAsSb Epilayers Grown by Molecular Beam Epitaxy
Source: Sci Rep. 2016 Jul 6;6:29112. doi: 10.1038/srep29112 (PMC4933967; doi:10.1038/srep29112)
Supplement: Supplementary Information [file srep29112-s1.pdf]

# Investigation of Localized States in GaAsSb Epilayers Grown By Molecular Beam Epitaxy

Xian Gao[1], Zhipeng Wei[1*], Fenghuan Zhao[2], Yahui Yang[2], Rui Chen[2*], Xuan Fang[1], Jilong Tang[1], Dan Fang[1], Dengkui Wang[1], Ruixue Li[1], Xiaotian Ge[1], Xiaohui Ma[1], Xiaohua Wang[1]

# Supplementary Information

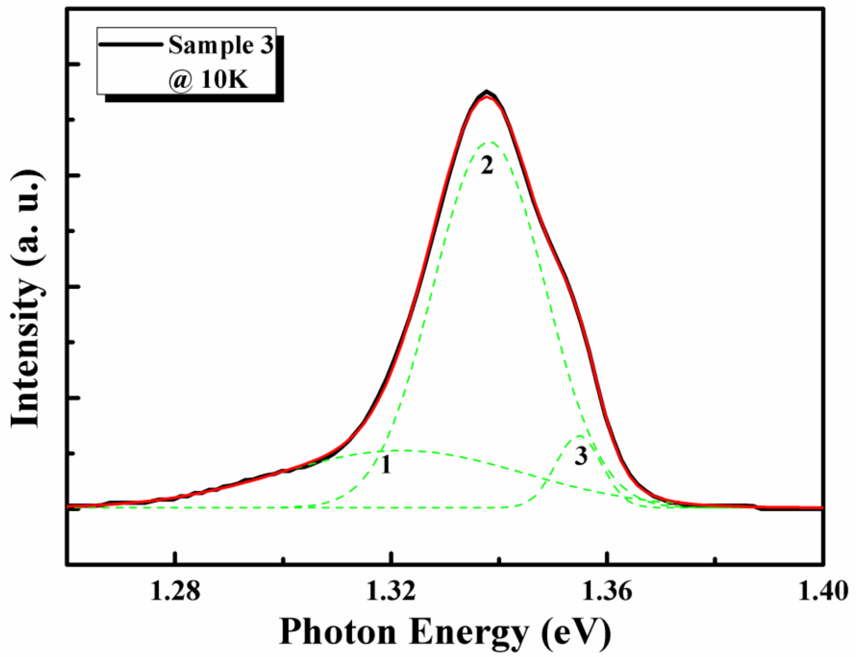


Supplementary Figure S1. The PL spectrum of sample 3 at 10 K and it could be deconvoluted into 3 emission peaks by using Gaussian fanction which shown by green dotted lines and marked as 1, 2 and 3, respectively.

Supplementary Table S1. the detailed information about these emission peaks.

| Sample 3 | Center (eV) | Area | Width (eV) |
|---|---|---|---|
| **Peak 1** | 1.304 | 59946 | 0.026 |
| **Peak 2** | 1.337 | 487640 | 0.021 |
| **Peak 3** | 1.355 | 55773 | 0.011 |

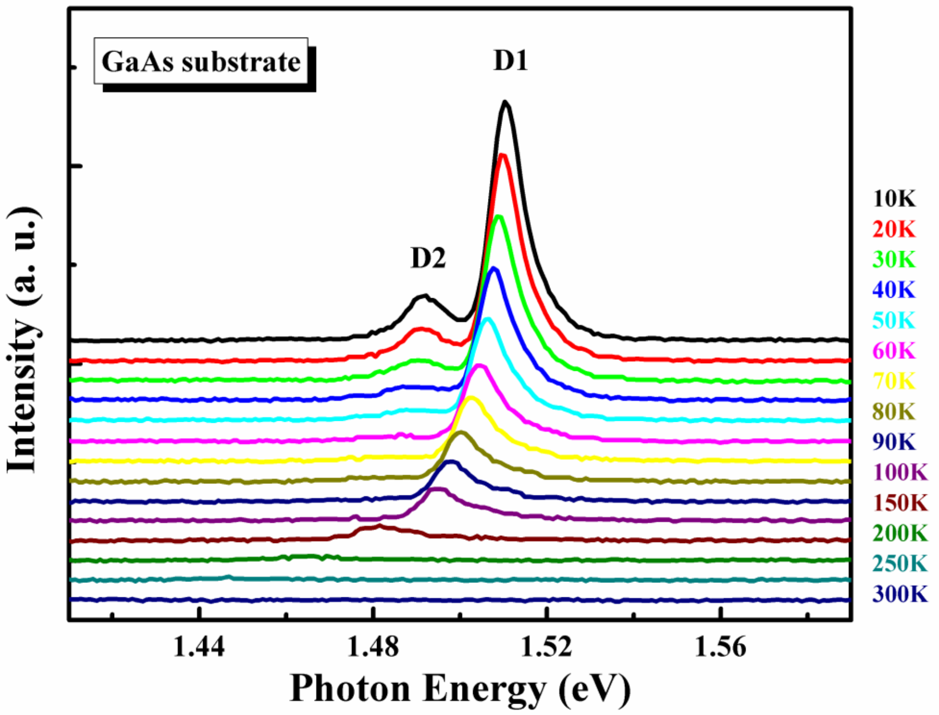


Supplementary Figure S2. Temperature dependent PL spectra of GaAs substrate.

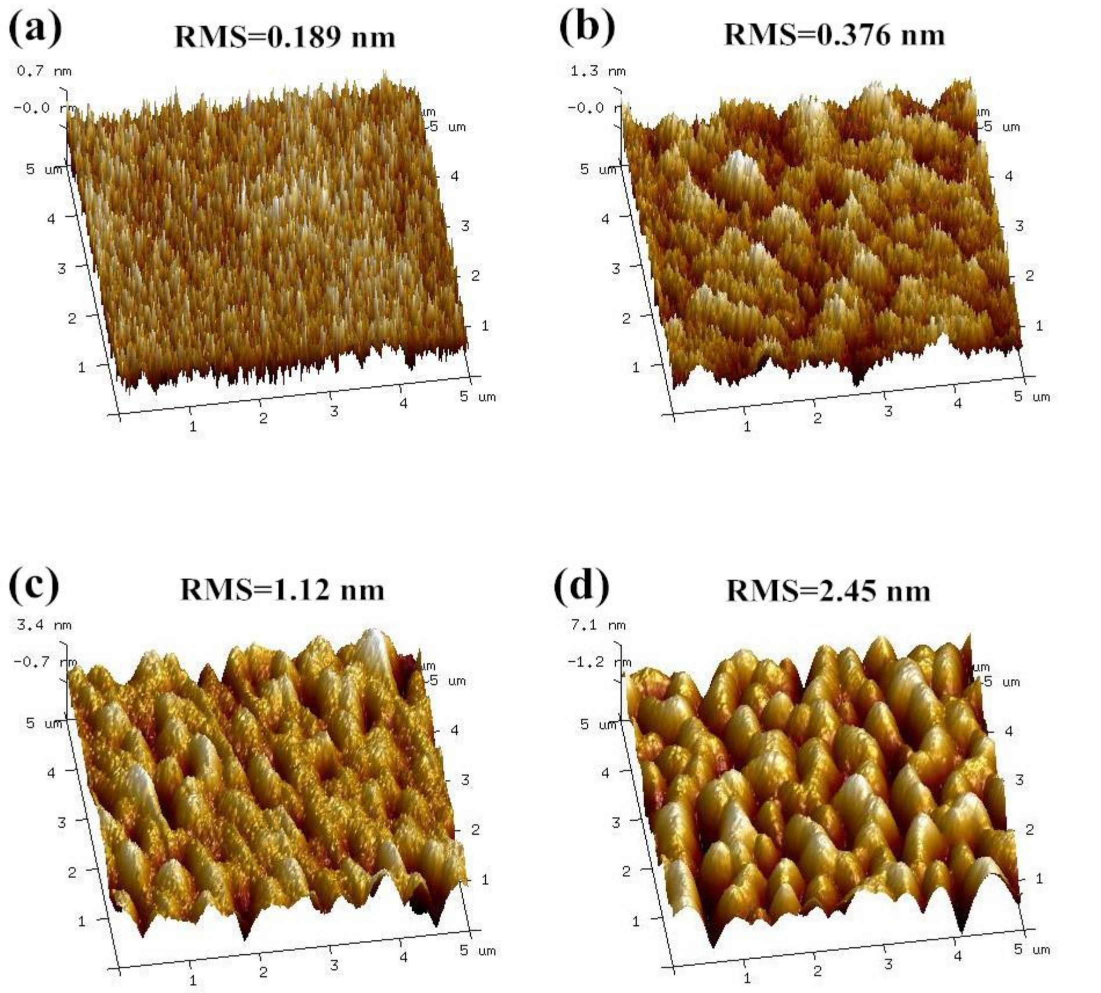


Supplementary Figure S3. AFM images of (a) GaAs substrate, (b) GaAsSb sample 1, (c) GaAsSb sample 2, and (d) GaAsSb sample 3 (RMS: Root Mean Squared roughness).

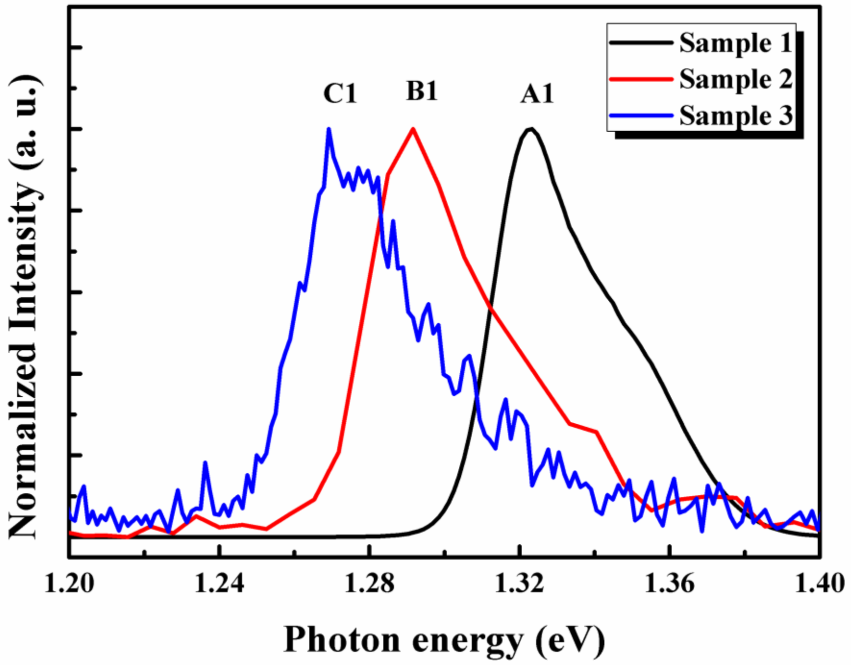


Supplementary Figure S4. The PL spectra of Sample 1, 2 and 3 at 300K, the location of the A1, B1 and C1 are 1.32, 1.29 and 1.27 eV, respectively.
